# Impact of Elevated Levels of Dissolved CO_2_ on Performance and Proteome Response of an Industrial 2′-Fucosyllactose Producing *Escherichia coli* Strain

**DOI:** 10.3390/microorganisms10061145

**Published:** 2022-06-01

**Authors:** Greta Gecse, André Vente, Mogens Kilstrup, Peter Becker, Ted Johanson

**Affiliations:** 1 HMO Innovation and Business Development, Royal DSM, Kogle Allé 4, 2970 Hørsholm, Denmark; greta.gecse@dsm.com (G.G.); p.becker@dsm.com (P.B.); 2 Department of Biotechnology and Biomedicine, Technical University of Denmark, Sølftofts Plads Building 221, 2800 Kgs. Lyngby, Denmark; mki@bio.dtu.dk; 3 Center for Analytical Innovation, Biodata & Translation, Science & Innovation, Royal DSM, Alexander Fleminglaan 1, 2613 AX Delft, The Netherlands; andre.vente@dsm.com

**Keywords:** scale-down, fermentation, physiology, *Escherichia coli*, proteome, dissolved carbon dioxide, 2′-focussyllatose, HMO, large-scale fermentation

## Abstract

Large-scale microbial industrial fermentations have significantly higher absolute pressure and dissolved CO_2_ concentrations than otherwise comparable laboratory-scale processes. Yet the effect of increased dissolved CO_2_ (dCO_2_) levels is rarely addressed in the literature. In the current work, we have investigated the impact of industrial levels of dCO_2_ (measured as the partial pressure of CO_2_, pCO_2_) in an *Escherichia coli*-based fed-batch process producing the human milk oligosaccharide 2′-fucosyllactose (2′-FL). The study evaluated the effect of high pCO_2_ levels in both carbon-limited (C-limited) and carbon/nitrogen-limited (C/N-limited) fed-batch processes. High-cell density cultures were sparged with 10%, 15%, 20%, or 30% CO_2_ in the inlet air to cover and exceed the levels observed in the industrial scale process. While the 10% enrichment was estimated to achieve similar or higher pCO_2_ levels as the large-scale fermentation it did not impact the performance of the process. The product and biomass yields started being affected above 15% CO_2_ enrichment, while 30% impaired the cultures completely. Quantitative proteomics analysis of the C-limited process showed that 15% CO_2_ enrichment affected the culture on the protein level, but to a much smaller degree than expected. A more significant impact was seen in the dual C/N limited process, which likely stemmed from the effect pCO_2_ had on nitrogen availability. The results demonstrated that microbial cultures can be seriously affected by elevated CO_2_ levels, albeit at higher levels than expected.

## 1. Introduction

**Figure 1:**
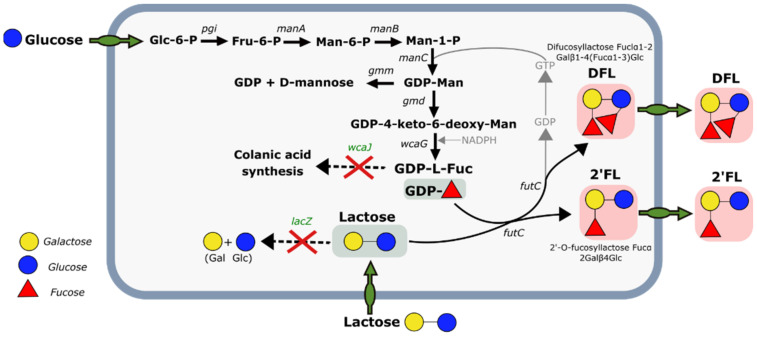
Simplified overview of the 2′-FL pathway in the *E. coli* production strain. The general modifications required for efficient 2′-FL synthesis include the expression of a heterologous fucosyl-transferase (encoded by futC), lacZ deletion to avoid breakdown of lactose, overexpression of the colanic acid pathway genes for efficient production of GDP-L-fucose, and the deletion of wcaJ to avoid further conversion of GDP-L-fucose to colanic acid. In addition to 2′-FL, the byproduct difucosyllactose (DFL) can be formed by the addition of a second fucose unit. Its formation rate is 2′-FL relative and dependent on the kinetics and rates of the reactions described above.

Human milk oligosaccharides (HMOs) constitute important and highly abundant components of mother’s milk that provide many health benefits to the neonate including the growth of beneficial gut bacteria and the improved function of the intestinal barrier [1,2,3]. Out of the HMOs in mother’s milk, 2′-fucosyllactose (2′-FL) is the most abundant [4] and therefore the most interesting from a commercial point of view. Today, 2′-FL is almost exclusively produced by fermentation where it is formed in vivo by the decoration of lactose with fucose through the action of a heterologous fucosyl transferase (Figure 1). *E. coli* has been the organism of choice for 2′-FL biosynthesis from the very beginning. In addition to being a well-known and easily modifiable workhorse in industrial biotechnology, it has the advantages of having a native lactose uptake system and a native colanic acid pathway to produce the activated GDP-L-fucose required for the fucosyl transferase reaction. Using *E. coli* as production host fermentations with 2′-FL titers of up to 180 g/L has been reported [5].

Fermentation based biomanufacturing has enabled 2′-FL production in *E. coli* at large industrial scales and it is now routinely produced in fermentation vessels of 200–400 m^3^ [5,6], where scale-dependent parameters play an important role and often create unexpected challenges. The long mixing times resulting from such enormous scales can be in excess of 60 s and lead to the formation of gradients in the substrate [7], dissolved oxygen [8,9] and pH [10], which in turn can affect the overall performance of the process [11]. In addition, the hydrostatic pressure together with a typically increased operating pressure increases the solubility of gasses such as O_2_ and CO_2_ [12]. Of these gasses, CO_2_, which has a relatively high solubility, is known to have negative effects on the stability, yield, and productivity of microbial processes when accumulating to high levels [13,14]. The level of a dissolved gas such as CO_2_ is often quantified by its partial pressure above the liquid (pCO_2_). This measurement is an approximation based on a proportional relation between the partial pressure of the gas and its dissolved level as described by Henry’s law [15].

**Figure 2:**
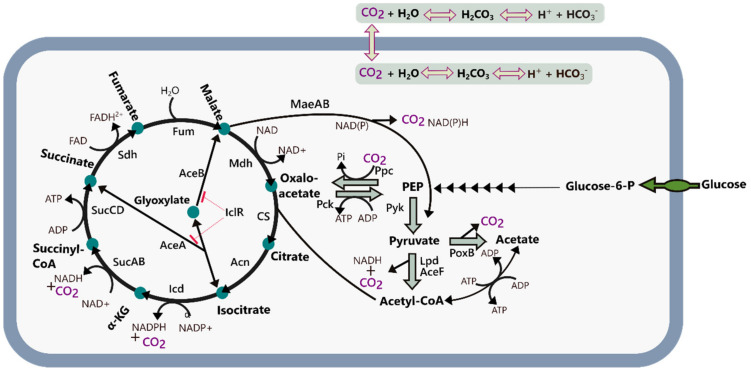
Schematic overview of the central carbon metabolism of E. coli including the CO_2_ producing/consuming reactions. Abbreviations: α-KG: α-ketoglutarate; Icd: isocitrate dehydrogenase; Acn: aconitate hydratase; GltA: citrate synthase; IclR: isocitrate lyase repressor; Mdh: malate-dehydrogenase; Fum: fumarase; Sdh: succinate:quinone oxidoreductase; SucCD: succinyl-CoA synthetase; SucAB: 2-oxoglutarate decarboxylase; PEP: phosphoenolpyruvate; Pyk: pyruvate kinase; Lpd: lipoamide dehydrogenase; AceF: pyruvate dehydrogenase; PoxB: pyruvate oxidase; Ppc: phosphoenolpyruvate carboxylase; Pck: phosphoenolpyruvate carboxykinase; MaeAB: malate dehydrogenase (oxaloacetate-decarboxylating).

While many studies have addressed the effect of the various gradients arising from the size of industrial vessels focusing both on appropriate scale-down model development and physiological characterization [8,9,10,16,17], only a handful have dealt with the impact of high pCO_2_ levels in *E. coli* processes [18,19,20,21]. Among these, only Knoll et al. have reported on a substrate-limited fed-batch process [21], which is the preferred mode of operation in fermentation-based manufacturing. The underlying mechanisms of how CO_2_ impacts fermentation performance are manifold. The kinetics of fundamental carboxylase and decarboxylase reactions that interconnect cellular anabolism, catabolism, and energy metabolism are directly affected by pCO_2_ (Figure 2). Direct toxic effects on membranes, cell structures, and proteins have also been reported [22,23,24,25], and since dissolved CO_2_ is in equilibrium with carbonic acid and bicarbonate it also acidifies the broth. CO_2_ thereby affects both osmolarity and pH since it triggers the addition of a titrant in pH-controlled fermentations. Increased pCO_2_ levels can therefore result in physiological effects stemming from osmotic pressure changes, pH changes, or by the direct impacts of pCO_2_ itself [19,26,27,28].

Since very little has been published on the impacts of pCO_2_ in fed-batch processes, the effects on the performance, physiology, and the proteome resulting from an extended exposure to the pCO_2_ levels typically encountered in industry are so far largely unknown or kept secret.

With this study we aimed to characterize the impact of elevated pCO_2_ on an *E. coli* based industrial fermentation process. Product yields were determined at different CO_2_ enrichment levels and compared to the performance in large-scale operations. The global proteome levels of the laboratory-scale runs were then studied under selected conditions. In addition, as the industrial process was limited on both carbon and nitrogen, the impact of enriched pCO_2_ on a C/N-limited process was evaluated and compared to a C-limited process to isolate the impact of CO_2_ from any potential differences in nitrogen limitation. To the best of our knowledge, this work shows the impacts of pCO_2_ on process performance and bacterial physiology in a high yielding industrial fed-batch process for the first time.

## 2. Materials and Methods

### 2.1. Strain

The strain used in all experiments was derived from *E. coli* K12 DH1 with the genotype: *F*^*-*^, *ʎ-*, *gyrA96*, *recA1*, *relA1*, *endA1*, *thi-1*, *hsdR17*, *supE44*. Additional modifications were made to generate the Strain 0: (i) deletion of *lacZ* to abolish β-galactosidase activity and prevent hydrolysis of lactose to glucose and galactose; (ii) deletion of the galactoside O-acetyltransferase gene *lacA*, which encodes for an enzyme that acetylates the galactose residues of oligosaccharides and would thereby lead to increased carbohydrate-type impurities; (iii) deletion of the *wcaJ* gene that encodes a lipid carrier transferase involved in colanic acid biosynthesis (colanic acid is an extracellular polysaccharide containing fucose and its overproduction dramatically increases the viscosity of the culture medium and acts as a drain on GDP-L-Fucose); (iv) deletion of the glucan biosynthesis glucosyltransferase H gene *mdoH* (the MdoH enzyme is involved in the biosynthesis of periplasmic glucans, the presence of which complicates the isolation and purification of targeted oligosaccharides); (v) deletion of the transcriptional repressor *glpR* to achieve higher expression levels of the genes controlled by the modified PglpF promoter used for 2′-FL synthesis; (vi) deletion of the lactose repressor *lacI* to remove the need for addition of isopropyl β-D-1-thiogalactopyranoside to induce expression of the Plac promoter controlled lactose permease encoded by *lacY*. Strain 0 was further engineered to generate the 2′-FL producing strain (Figure 1) used for the experiments by chromosomally integrating two copies of the alpha-1,2-fucosyltransferase *futC* from *Helicobacter pylori* 26,695 (homologous to NCBI Accession nr. WP_080473865.1 with two additional amino acids (LG) at the C-terminus) under the control of a modified PglpF promoter [29], and an additional copy of the colanic acid operon (*gmd-wcaG-wcaH-wcaI-manC-manB*) under the control of the same modifed PglpF promoter. The modified PglpF promoter and the Plac promoter, sans *lacI*, were both automatically induced in the absence of catabolite repression. Thus, high level expression of the colanic acid genes, *futC* and *lacY*, were initiated when the cultures transitioned from catabolically repressed exponential growth in the batch phase into glucose limited growth in the fed-batch phase. Cryovials containing the strain in 25% (*v*/*v*) glycerol solution were stored at −80 °C prior to use.

### 2.2. Precultures

Precultures were prepared in two steps and were cultivated overnight at 33 °C with 200 rpm shaking. The frozen *E. coli* stock was used to inoculate a preculture with 10 mL minimal glucose media in a 50 mL Falcon tube. The medium was composed of 10 g/L NH_4_H_2_PO_4_, 5 g/L KH_2_PO_4_, 1 g/L citric acid, 2.35 g/L NaOH, 1.65 g/L KOH, 5 g/L K_2_SO_4_, 10 g/L trace metal solution, and finally 1 g/L MgSO_4_.7H_2_O and thiamine solutions, which were sterilized and added separately. A second preculture in a 250 mL baffled shake flask with 50 mL of the same minimal glucose medium was inoculated with the overnight culture to a final optical density at 600 nm (OD_600_) of 0.25. The shake flask was then incubated at 33 °C and 200 rpm for 6–9 h until a final OD_600_ of 3–5 and thereafter used to inoculate the main culture.

### 2.3. Fed-Batch Bioreactor Cultivations

Lab-scale fermentations were carried out in 2 L Sartorius Biostat B fermenters equipped with an MFCS-monitoring system (Sartorius). The fermentations were glucose limited fed-batch processes using the same minimal medium composition and feed profile as the large-scale process (confidential) with a starting mass of 1.2 kg. Main cultures were inoculated with liquid precultures to a 2% (*v*/*v*) final ratio. Both the fermentation media and the feed contained glucose and lactose. The DO was controlled by a stirring (700–2000 rpm) and airflow (1–3 VVM) cascade set to 23%. The pH level was kept at 6.8 by NH_4_OH titration. The system was equipped with pO_2_ (Hamilton), pCO_2_ (only for a few experiments, iSense 5000i, Mettler and Toledo, Columbus, OH, USA), temperature, and pH (Hamilton) sensors. Statistical analysis of fermentation data was performed in SAS JMP.

### 2.4. CO_2_ Enrichment

In the CO_2_ enriched fermentations, pure CO_2_ was added to the airflow inlet and was initiated when the fed-batch phase started. The CO_2_ concentration in the inlet air was kept constant by an airflow controller. The pCO_2_ levels were either monitored with a probe or estimated by superimposing the pCO_2_ in the inlet gas stream with the pCO_2_ levels that were measured in the reference fermentations without enrichment. An overview of all the fermentations performed in this study is presented in Table A1 in the Appendix A.

### 2.5. Calibration of CO_2_ Probe

As a proof of concept, the level of pCO_2_ was monitored with a probe (i5000 Mettler and Toledo). The probe was autoclaved in a separate vessel with a batch phase fermentation mineral medium, as described above. After sterilization, the pH was adjusted to 6.8 by NH_4_OH, the temperature was set to 33 °C, and the stirring was set to 700 rpm. A two-point calibration was performed by measuring the pCO_2_ level after sparging media with gas mixtures of 20/80% CO_2_/N_2_ and 8/10/82% CO_2_/O_2_/N_2_. The calibration process was monitored using i5000 software and saturation was assumed when the values became stable. The probe was then moved to the fermenter used for the experiments inside a sterile laminar flow bench to avoid contamination. In the industrial scale fermenter, the same process calibration was performed before the probe was mounted in the fermenter prior to sterilization.

### 2.6. Sampling and Analytical Procedures

The 2′-FL, DFL, and lactose levels in the fermentation broth samples were quantified by HPLC. Samples taken from the vessel were immediately diluted with deionized water and boiled for 20 min. After the heat-treatment, the samples were centrifuged for 3 min at 17,000× *g* and the resulting supernatant analyzed by HPLC (Dionex Ultimate 3000 RS, Thermo Scientific, Waltham, MA, USA) using a Supelco TSK gel Amide-80 HPLC column with a 68% acetonitril isocratic solvent. The biomass was monitored as bio wet mass (BWM), defined as the weight ratio of the pellet to the pellet and the supernatant after 3 min centrifugation at 17,000× *g*. The BWM values were converted into dry cell weight (CDW) using a ratio that was determined in previous experiments (data not shown). Samples for acetate measurements were taken by a 3 mL syringe (HENKE-JECT^®^, Henke, Sass, Wolf GmbH, Tuttlingen, Germany) and were directly filtered through a 0.45 µm cellulose syringe filter (30 mm diameter, Thermo Fisher, Waltham, MA, USA). NH_4_^+^ and phosphate levels were estimated from supernatant samples by using Quantofix^®^.

### 2.7. Proteomics Analysis

Cells were harvested at different time points after feed start in the fed-batch process (6, 30, 80, 120 h). The precise timepoints of each condition are listed in Appendix A, Table A1. Fermentation broth was sampled directly into a syringe filled with ice cold 0.9% NaCl solution which diluted the broth approximately 3-fold. The syringe was measured before and after adding the fermentation broth to calculate the dilution factor and the cell weight. The solution was kept on dry ice until transport to the centrifuge. The solution was centrifuged at 4100× *g* for 10 min at 4°C. The pellet was then washed in ice-cold 0.9% NaCl solution and pelleted again by centrifugation for 5 min at 6000× *g* at 4 °C. The pellet was then immediately placed on dry ice and transferred into a −80 °C freezer where it was stored until the analysis.

Proteome analysis was performed at the DSM Biotechnology Center in Delft, NL. Lysis buffer (PreOmics) was added to the frozen cell pellets and the solutions heated for 15 min at 95 °C. For proteomics analysis, lysates were normalized to an equivalent of 10 mg lysed cells followed by reduction, alkylation, and digestion using trypsin. Samples were analyzed in technical triplicates by liquid chromatography tandem mass spectrometry (LC-MS/MS) using a Vanquish UHPLC coupled to a Q Exactive Plus Orbitrap MS (Thermo Fisher Scientific). Peptides were separated via reverse-phase chromatography using a gradient of water with 0.1% formic acid (solvent A) and 80% acetonitrile with 0.1% formic acid (solvent B) from 5% B to 40% B in 20 min with a flow rate of 400 µL/min. Data-independent acquisition (DIA) was performed with a resolution setting at 17,500 within the 400- to 1200-*m*/*z* range and a maximum injection time of 20 ms, followed by 8 high-energy collision-induced dissociation activated (HCD) MS/MS scans with a resolution setting at 17,500 covering the mass range from 400 to 875 *m*/*z* using 60Da collision windows.

Data was analyzed with Spectronaut version 14.10 (Biognosys, Schlieren, Switzerland) [30], using the direct DIA approach with a protein database for the specific strain used in the study allowing Trypsin/P specific peptides including 2 missed cleavages, an oxidation on methionine, carbamidomethylated cysteines, and deamidated asparagine and glutamine. Label-free quantification was performed using the top three unique peptides measured for each protein. Retention time alignment was performed on the most abundant signals obtained from peptides measured in all samples, and results were filtered by FDR of 1% followed by normalization of the result using the median ion intensities measured for each sample.

### 2.8. Data Analysis

Proteomics data analysis and differential expression analysis (DEA) were performed in R, using a custom-made package based on limma in R. The protein counts were centralized, and DEA was used to compare the protein expressions at given time points between control and CO_2_ enriched conditions. Three different time points were compared from the C-limited fermentations: 6, 30, and 120 h after feed start and two in the C/N-limited fermentations 30 and 120 h after feed start.

The DEA analysis was performed in R using the limma package with linear models. To determine the differentially expressed proteins, standard filtering conditions based on fold change (FC) and significance were used with the following settings: FC > 1.5, *p* < 0.05.

## 3. Results

### 3.1. pCO_2_ Levels in Industrial and Laboratory Scale

**Figure 3:**
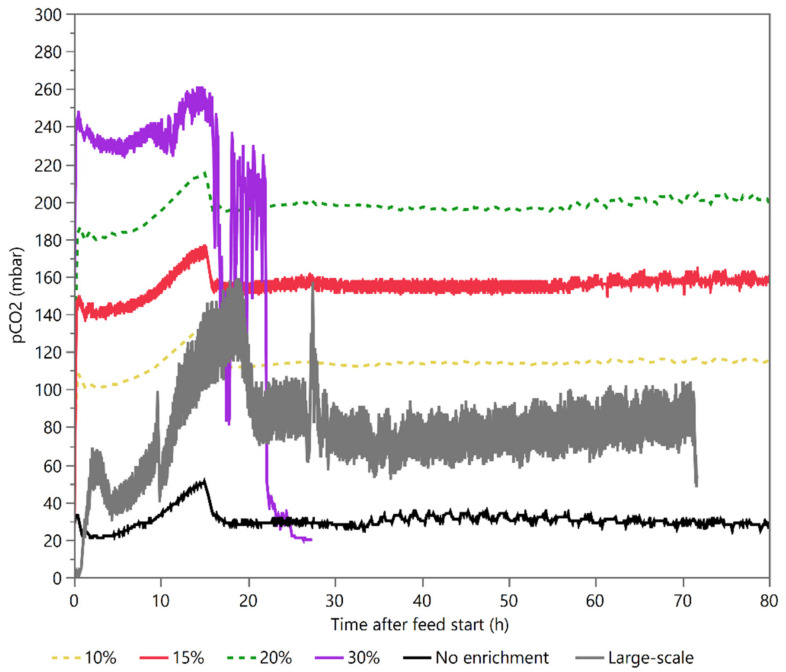
Measured and estimated pCO_2_ concentrations. pCO_2_ concentrations were measured from fermentations with 15% CO_2_ enrichment (red), 30% CO_2_ enrichment (purple), non-enriched control fermentations (black), and a large-scale fermentation (grey). pCO_2_ values from 10% (yellow dashed line) and 20% (green dashed line) enrichments were not measured but estimated from the measurements from the 15% and the control fermentations. The estimated pCO_2_ curves are shown as 20 min moving averages of measured pCO_2_ data with superimposed values from the inlet air stream to reflect the actual enrichment.

The pCO_2_ levels of an industrial 2′-FL process performed in a 450 m^3^ fermentation vessel were measured by a commercial probe located close to the bottom of the vessel and compared to a corresponding laboratory process. As expected, the measured pCO_2_ levels were significantly higher in the large fermenter with a two–three-fold increase and a peak level of 150–160 mbar (Figure 3). These levels were in a range where previous studies had reported negative effects on *E. coli* cultures, albeit in a batch growth [19]. Follow-up runs using scale-down models with 15% and 30% CO_2_ enrichment in the inlet sparging gas were also measured with the CO_2_ probe. In addition, scale-down runs with 10% and 20% enrichment were carried out without a probe but had their pCO_2_ levels estimated. The measured and estimated pCO_2_ levels can be seen in Figure 3.

### 3.2. Dual Limitation in the Fermentation Process

**Figure 4:**
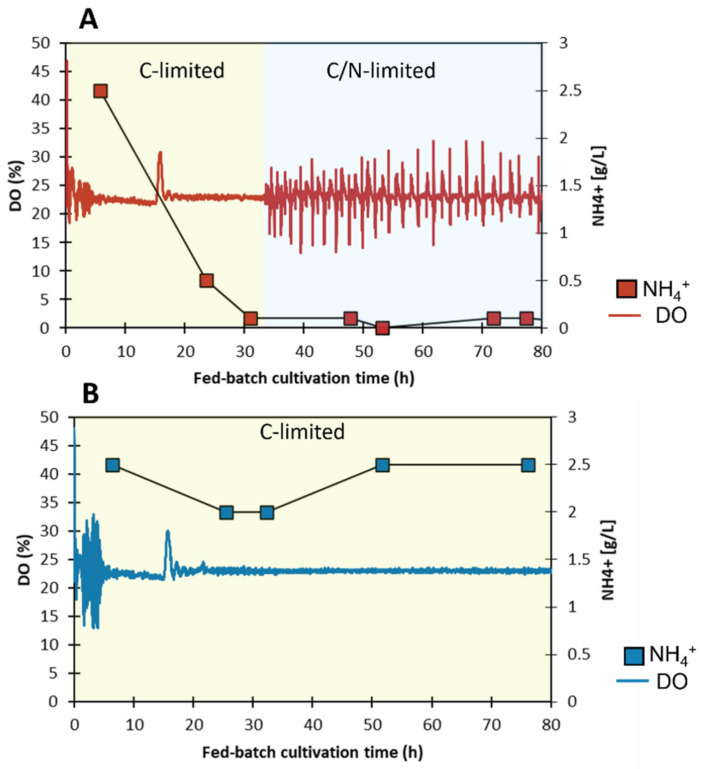
Baseline laboratory-scale fed-batch processes without CO_2_ enrichment. (**A**) C/N-limited process. N-limitation began approximately 30 h after the feed started manifesting as low extracellular NH_4_^+^ concentrations and metabolic oscillations in DO, pH, CO_2_, and other parameters. DO levels are presented as an example. The C-limited phase is marked with yellow and the dual C/N-limited phase with light blue. (**B**) Sole C-limitation was achieved by supplementation with ammonium sulfate. NH_4_^+^ concentrations are shown with square markers.

The fermentation process in this study had an unusual trait. While initially being C-limited, the process naturally became N-limited approximately 30 h into the fed-batch phase, whereafter it settled into an oscillatory state seemingly shifting back and forth between N-limitation coupled with a slight overflow metabolism and pure C-limitation. This behavior was observed in both large- and laboratory-scale processes and its onset could be observed by following the [NH_4_^+^], but also by the emergence of obvious oscillations of the on-line parameters such as the pH, CO_2_ evolution, and dissolved oxygen levels (see example in Figure 4A).

**Figure 5:**
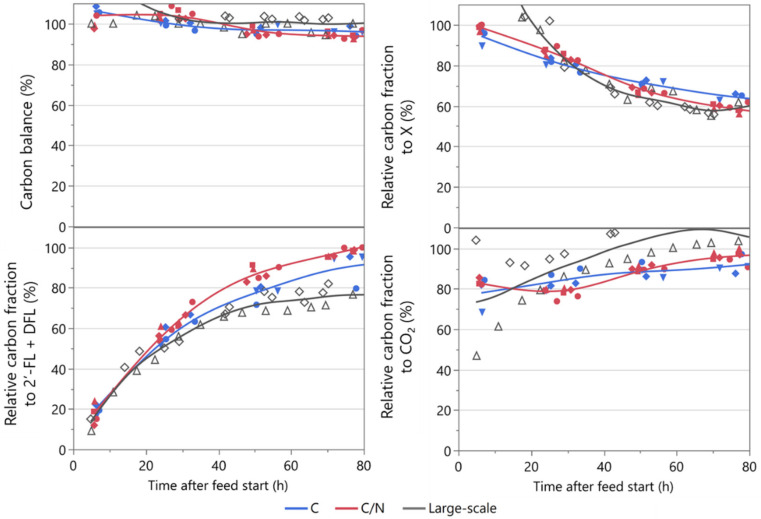
Carbon balance and carbon allocation of laboratory and industrial fermentations. Carbon allocation to the products 2′-FL and DFL as well as biomass (X) and CO_2_ were estimated from offline and online measurements. Each curve represents three independent experiments from laboratory-scale and two from large-scale processes. The values were relativized by normalizing to the end-point value of the average of the three fermentations without CO_2_ enrichment under C/N limitation.

As N-limitation and C-limitation have very different regulations of gene expression, [31] any change to the degree of N-limitation was expected to have a profound impact on the physiology of the *E. coli* strain. This could include shifts in maintenance requirements, metabolic pathway regulation, and the transcriptome and proteome profiles, which in turn could lead to shifts in biomass and product yields. Since the pCO_2_ level affects pH and thereby indirectly the N-level via the pH titrant NH_4_OH, it could potentially reduce or even relieve the impact of N-limitation and thereby obscure other effects caused by increased pCO_2_. To introduce a control for this potential bias, laboratory cultivations with and without pCO_2_ enrichment with excess nitrogen were also included in the study. In these fermentations, additional NH_4_^+^ was supplemented via the base titrant in the form of (NH_4_)_2_SO_4_ to keep the [NH_4_^+^] between 2–3 g/L in the fermenter (Figure 4). Having extra nitrogen available for growth also led to a higher biomass (Figure 5) after 50 h of the fed-batch phase. A carbon allocation comparison showed that the carbon from this extra biomass was predominantly taken from the product formation, whereas the CO_2_ evolution was similar (Figure 5). While the allocation to product formation at a large-scale was initially similar to either C- or C/N-limited laboratory-scale fermentations, it became significantly lower and had a slightly lower biomass after the onset of C/N-limitation. Instead, CO_2_ evolution was much higher.

### 3.3. Fermentation Performance with and without pCO_2_ Enrichment

To investigate the impact of high pCO_2_ levels, both C- and C/N-limited fermentations were compared at different levels of CO_2_ enrichment in at least two independent fermentations. Product yields were evaluated as the sum of the carbon allocated to the produced HMOs (2′-FL + DFL) per glucose added. The summary of the 15 fermentations performed during this study is presented in Table 1.

**Figure 6:**
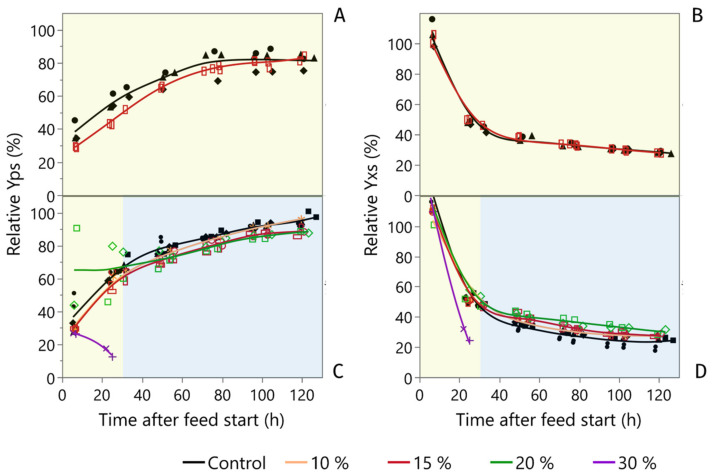
Relative product and biomass yields on glucose. (**A**) Product yield (Yps-2′-FL + DFL) and (**B**) biomass yield (Yxs), under C-limitation, (**C**) product yield and (**D**) biomass yield under C/N-limitation with different levels of CO_2_ enrichment. Different marker types represent distinct replicate fermentations. The values were relativized by normalizing to the end-point value of the average of the three fermentations without CO_2_ enrichment under C/N limitation.

**Figure A1:**
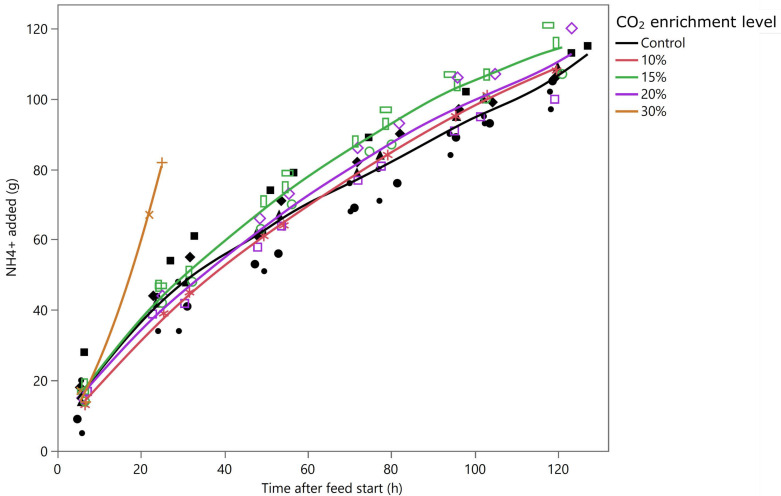
Ammonium (NH_4_^+^) addition for C/N condition with different CO_2_ enrichment levels for experiments with C/N limitation. The gravimetric NH_4_^+^ addition was calculated from the base addition consisting of NH_4_OH. Each symbol represents a distinct experiment.

The fermentation that had the closest pCO_2_ level to large-scale fermentations was the 10% CO_2_ enrichment, which was approximately similar or higher in pCO_2_. (Figure 3). However, this enrichment did not lead to any observable difference in the performance as measured in the product and biomass yields (Table 1, Figure 6C,D). Increasing the enrichment to 15% pCO_2_ led to a slightly increased biomass yield for the C/N limited process and increasing the enrichment further to 20% pCO_2_ led to an additional biomass increase (Table 1, Figure 6C,D). The pCO_2_ enrichment first had an impact on biomass after 40 h, which corresponded with the onset of N-limitation. In addition to biomass, product yields were also affected at 15% and 20% pCO_2_. Again, this effect started at the onset of N-limitation. A further increase of pCO_2_ to 30% caused a marked increase in the base consumption right from the onset of the enrichment and led to a complete loss of culture viability approximately 20 h later (Figure 6C,D). A sample taken at 25 h of fermentation revealed 32.5 g/L acetic acid and 3.7 g/L glutamic acid, showing that the much larger base pull (Appendix C, Figure A1) was caused by acid accumulation.

The 15% pCO_2_ enrichment was selected as the focus for the proteomics study and the C- vs. C/N comparison even though it had a higher average pCO_2_ level than what was measured in the large-scale fermentation (Figure 3). This decision was taken since it was the lowest pCO_2_ level that had a discernible impact on the fermentation. It was therefore considered to have a higher likelihood of undergoing a physiological change that could be resolved in the proteomics data and was still at a level with industrial relevance. In the C-limited study, no impact on biomass yield could be seen with the 15% pCO_2_ enrichment (Figure 6B). The enrichment initially led to a lower product yield, but this difference diminished and eventually disappeared over time (Figure 6A). It should be noted that the deviation was high between the three replicates in the control group caused by a potential outlier (Figure 6A, black full diamonds). C-limitation on its own decreased the product yield compared to the regular C/N-limited process with and without CO_2_ enrichment. (Table 1). 

### 3.4. Proteome Analysis

A timeseries proteomics study was conducted to evaluate the effect of pCO_2_ enrichment under C- and C/N limited conditions. The samples from the enriched processes were compared to their respective 0% references to gain an overview of the impact of elevated pCO_2_ levels on the bacterial physiology. For the 15% pCO_2_ enrichment analysis, cells were harvested from three or four time points in at least duplicate fermentations. The 10% and 20% pCO_2_ enriched fermentation data was derived from single experiments; however, the data in general were very reproducible and therefore single determinations were still included in the data analysis. In the study, which was not optimized for membrane proteins, a total of 1546 proteins were detected.

#### 3.4.1. Identification of Differentially Expressed Proteins

**Figure 7:**
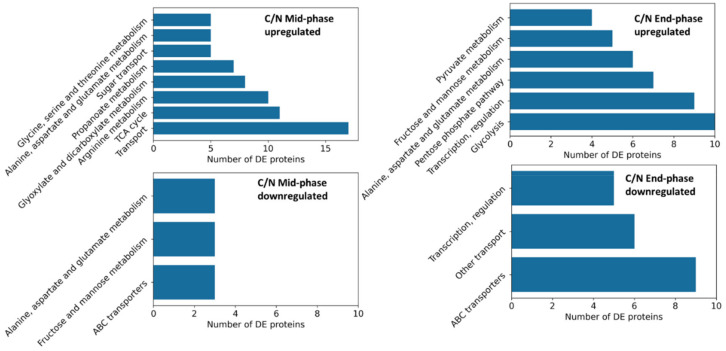
GO enrichment of differentially expressed proteins of 15% versus 0% pCO_2_ enriched cultures under C/N-limited conditions.

The number of significantly differentially expressed (DE) proteins between 0% and 15% CO_2_ enriched conditions at given timepoints are presented in Table 2. In general, there were higher numbers of differentially expressed proteins in the C/N limited condition. A functional Gene Ontology (GO) enrichment analysis was performed to find patterns and used to divide these differentially expressed proteins into groups (Figure 7). GO enrichment analysis revealed that the identified functional groups from the middle and late fermentation phases were similar. The highest-ranking groups were related to nitrogen and carbon metabolism and transportation. In the middle-fermentation phase, tricarboxylic acid (TCA) cycle and arginine biosynthesis related proteins were upregulated with CO_2_ enrichment, while transport and glutamate related proteins were downregulated. At the late fermentation stage, transport (especially ABC transporters) and glutamate related proteins were downregulated, whereas glycolysis and pyruvate metabolism related proteins were upregulated (Figure 7).

Far fewer differentially expressed proteins were identified in the C-limited condition (Table 2). It was therefore not feasible to quantitatively group the differentially expressed proteins based on their functions. In the mid-fermentation phase, many of the upregulated proteins were related to acid stress response, such as GadA, GlsA, and GadB. In the late fermentation phase, there were only five differentially expressed proteins left. Three proteins were upregulated with CO_2_ enrichment: Ada (3-fold), DmlA (1.7 fold), and EutM (1.5-fold); two were downregulated FlgM (2.5 fold) and DadA, (4 fold). Interestingly, flagellin synthesis-related proteins were downregulated in all timepoints. On the other hand, CsgD, curli operon transcriptional regulatory protein, LolB, outer membrane lipoprotein, and PspC phage shock proteins were all upregulated. These could potentially serve as a protection from the increased pCO_2_.

There were only a few identified proteins that were commonly changed for the C- and C/N-samples as a result of pCO_2_ enrichment. These proteins were: GlsA, DmlA, GltA and Mdh. Thus, malate dehydrogenase and glutamine/glutamate metabolisms were affected by the increased CO_2_ levels regardless of the nitrogen limitation.

#### 3.4.2. Time Course Expression Changes of 2′-FL Production and TCA Related Proteins

To investigate the underlying cause of the lower product yields in the enriched fermentations, the expression profiles of proteins involved in the 2′-FL production pathway, TCA cycle, and carboxylation reactions were compared under the different conditions. The targets are listed in Appendix B, Table A2.

**Figure 8:**
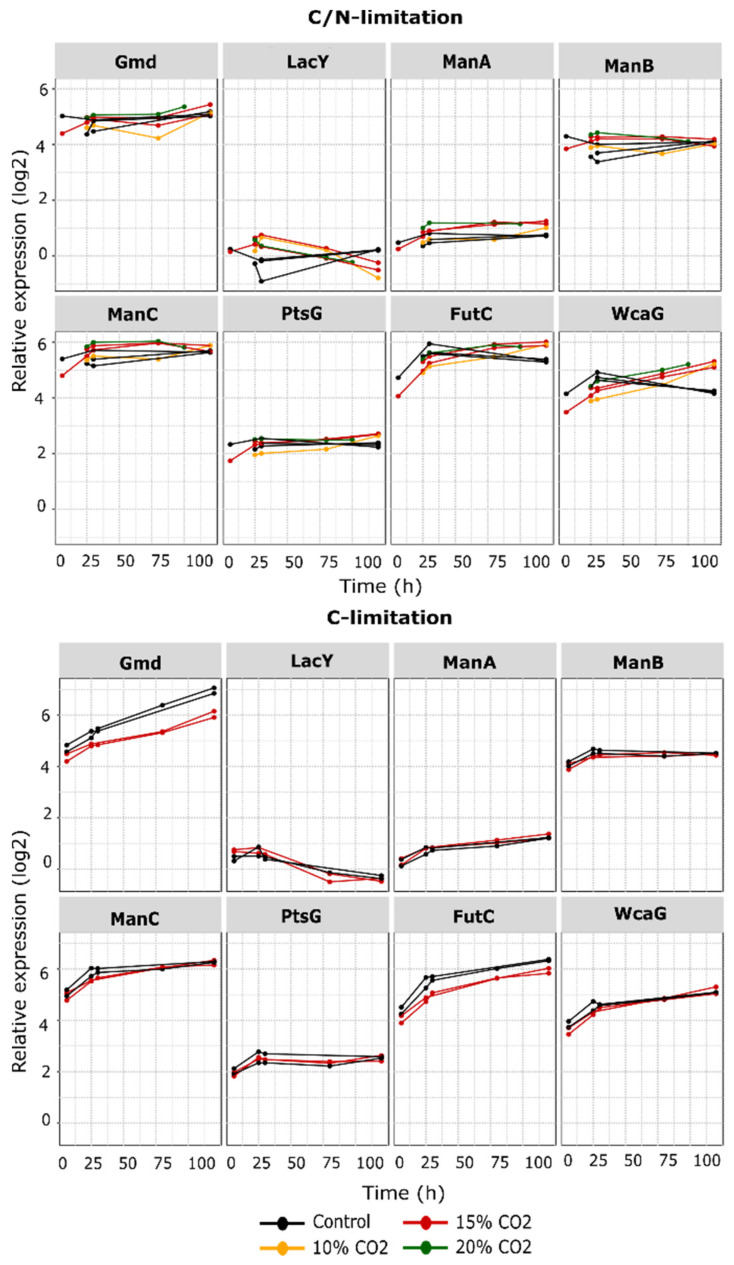
Expression profiles of proteins in the 2′-FL production pathway. *Y*-axis = relative expression in log2, *X*-axis = fermentation age after feed start (h). Gmd: GDP-mannose 4,6-dehydratase, LacY: lactose permease, ManA: mannose-6-phosphate isomerase, ManB: phosphomannomutase, ManC: mannose-1-phosphate guanyltransferase, PtsG: glucose-specific PTS enzyme IIBC component, FutC: alpha-1,2-fucosyltransferase, WcaG/Fcl: GDP-L-fucose synthase.

**Figure 9:**
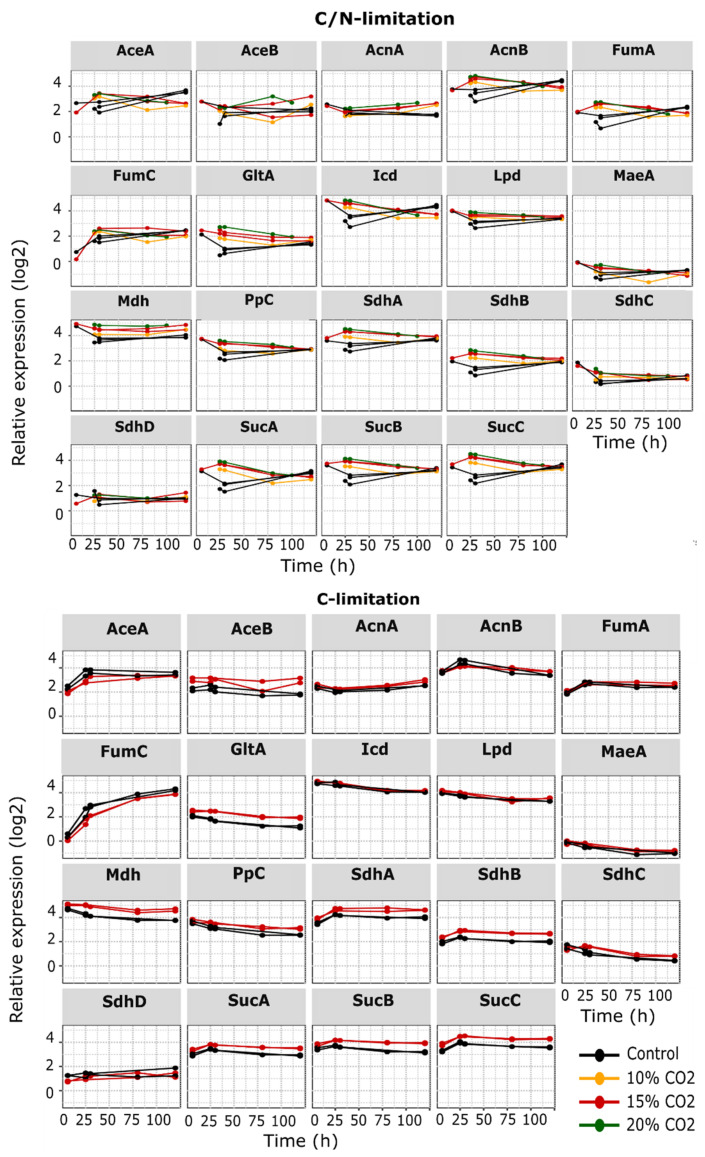
Protein expression level in the TCA cycle under different fermentation conditions: C/N- limited with 0%, 10%, 15%, and 20% pCO_2_ enrichment and C-limited with 0% and 15% pCO_2_ enrichment. *Y*-axis = relative expression in log2, *X*-axis = fermentation age after feed start in hours. AceB: malate synthase A, AceK: isocitrate dehydrogenase kinase, AcnA: aconitate hydratase A, AcnB: aconitate hydratase, FumA: fumarase A, FumC: Fumarate hydratase, GltA: citrate synthase, Icd: Isocitrate dehydrogenase, Lpd: lipoamide dehydrogenase, MaeA: malate dehydrogenase, Md: malate dehydrogenase, Ppc: Phosphoenolpyruvate carboxylase, SdhA: succinate:quinone oxidoreductase, SdhB: succinate:quinone oxidoreductase, SdhC: succinate:quinone oxidoreductase, SdhD: succinate:quinone oxidoreductase, SucA: 2-oxoglutarate decarboxylase, SucB: dihydrolipoyltranssuccinylase, SucD: succinyl-CoA synthetase subunit.

**Figure 10:**
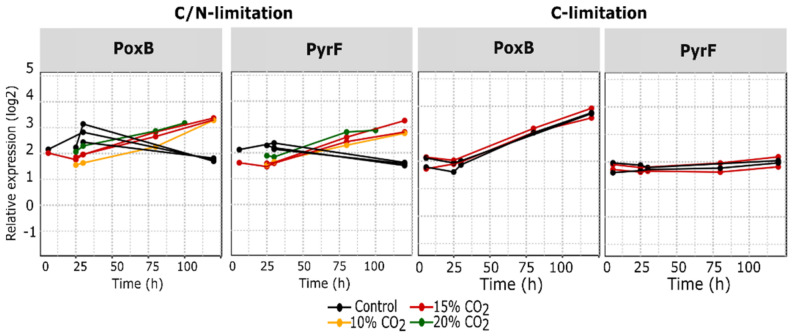
Changes in expression level of selected proteins involved in carboxylation and decarboxylation reactions. *Y*-axis = relative expression in log_2_, *X*-axis = fermentation age after feed start in hours. Ppc: Phosphoenolpyruvate carboxylase, PyrF: Orotidine 5′-phosphate decarboxylase, PoxB: Pyruvate oxidase.

In the proteome data, the C/N limited samples with 15% and 20% CO_2_ enrichment grouped together while the 10% samples were closer to the control group (Figure 8, Figure 9 and Figure 10). 

### 3.5. Proteins from the 2′-FL Production Pathways Were Not Significantly Affected by CO_2_ Enrichment

In general, the proteins directly involved with 2′-FL production were not drastically affected by the elevated pCO_2_ levels, regardless of the limitation state (Figure 8). In the C/N limited dataset, Fcl (WcaG), ManA, and FutC had a slightly higher abundance under CO_2_ enrichment at the late stage of the fermentation (Figure 8). Under C-limitation, a clear difference was observed in Gmd and FutC abundancy, which both had lower expressions throughout the fermentation (Figure 8).

### 3.6. CO_2_ Enrichment Increased TCA Cycle Protein Expression

In general, TCA related proteins were stably expressed under all tested conditions, but many of them were observed to be expressed at a higher level under CO_2_ enrichment (Figure 9). Although these patterns were highly reproducible, most of the fold changes did not reach the minimum threshold of abs|FC| = 1.5 in the DEA. Therefore, in strict terms, none of the TCA target proteins had a significantly changed expression under the C-limited condition. On the other hand, several TCA enzymes such as GltA, SucABC, and SdhAB were affected in the C/N limited condition.

### 3.7. Enzymes Involved in Carboxylation and Decarboxylation Reactions

It was hypothesized that pCO_2_ levels could affect the expression of enzymes in-volved in carboxylation and decarboxylation reactions. Therefore, a total of 11 proteins involved in carboxylation (Ppc and Psd) and decarboxylation (MaeAB, PoxB, SucAB, Icd, AceF, PyrF, Lpd, NadC, and HemE) reactions were specifically examined in the dataset.

**Figure A2:**
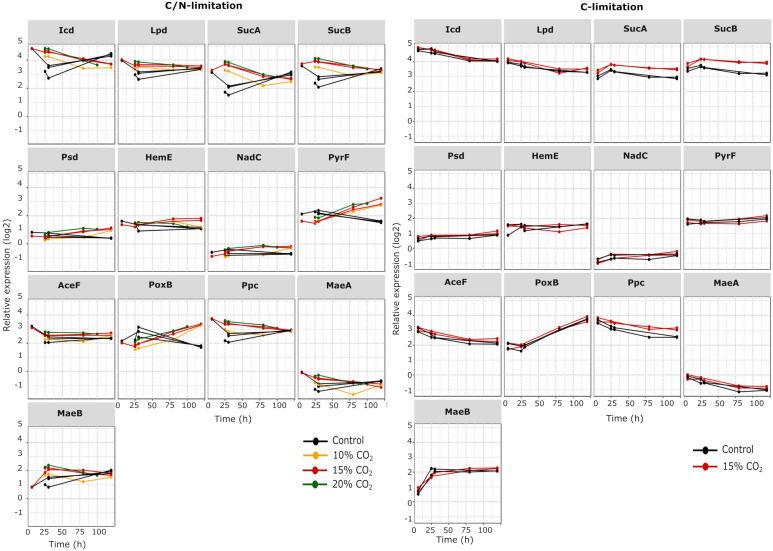
Changes in expression level of selected proteins involved in carboxylation and decarboxylation reactions. *Y*-axis = relative expression in log2, *X*-axis = fermentation age after feed start in hours. Ppc: Phosphoenolpyruvate carboxylase, AceF: Acetyltransferase, PyrF: Orotidine 5′-phosphate decarboxylase, NadC: Nicotinate-nucleotide pyrophosprolyase, decarboxylating, HemE: Uroporghyrinogen decarboxylase, Psd: Phosphatidylserine decarboxylase proenzyme, SucA: 2-oxoglutarate decarboxylase, SucB: succinyltransferase, LpdA: lipoamide dehydrogenase, Icd: isocitrate dehydrogenase, PoxB: Pyruvate oxidase, MaeA: malate dehydrogenase, oxaloacetate-decarboxylating, MaeB: malate dehydrogenase, oxaloacetate-decarboxylating.

CO_2_ enrichment under C-limited conditions led to higher SucAB expression throughout the fermentation process. However, this increase was also observed for the other proteins from the TCA cycle and is therefore not directly linked to changing decarboxylation kinetics (Figure 10). A similar pattern was observed under C/N limited conditions, but only until the middle part of the fermentation. At the late stage, the expression profiles of the CO_2_ enriched groups dropped and became similar to that of the control. On the other hand, PyrF, an orotidine-5′-phosphate decarboxylase catalyzing the last step in the pyrimidine synthesis, started low but had an increasingly higher expression after 30 h (Figure 10). This behavior was not observed in the C-limited dataset. The expression pattern of other decarboxylases such as Psd, HemE, and NadC were similar to PyrF in the C/N limited dataset but the differences between the conditions were not enhanced to the same extent (Appendix D, Figure A2). Surprisingly, except for Ppc, none of the targeted carboxylases were impacted by the pCO_2_ levels under solely C-limitation. For Ppc, the abundancy was higher with CO_2_ enrichment under both C and C/N limited conditions (Figure 10).

### 3.8. Nitrogen Uptake Proteins

**Figure A3:**
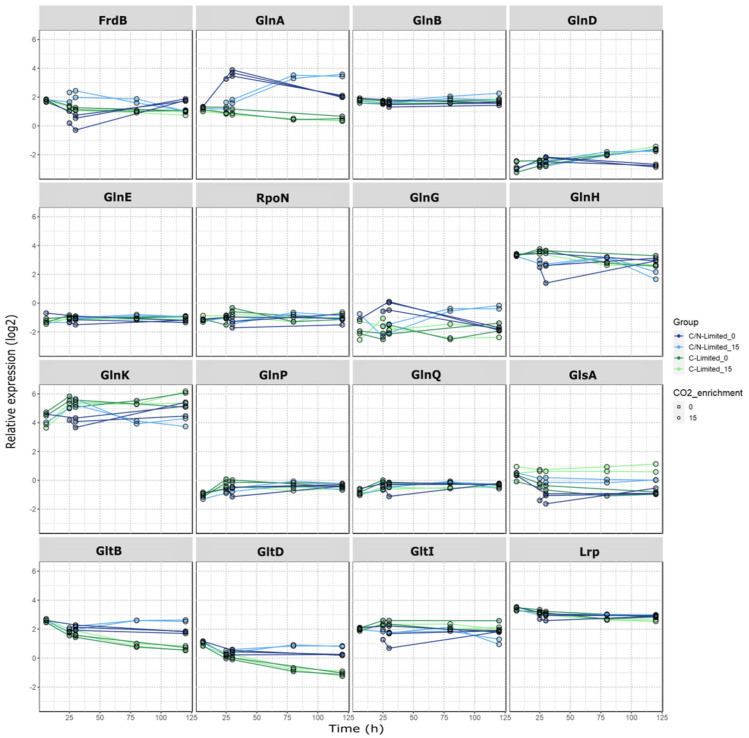
Expression profile of acid stress and nitrogen assimilation related proteins. *Y*-axis: relative expression in log2 of proteins involved in acid stress response and nitrogen assimilation. *X*-axis: fermentation time after feed start.

As expected, proteins involved in nitrogen assimilation were differentially expressed when comparing the C- and C/N-limited datasets. This was shown in the expression profiles of GlnA, GltB, and GltD (Appendix E, Figure A3). In the C/N limited condition, GlnA expression was increased at the timepoint when the culture reached ammonium limitation approximately 40 h into the fed-batch phase. It was also clear that the high expression of GlnA started later in the CO_2_ enriched samples showing that CO_2_ enrichment delayed the start of the nitrogen limitation.

## 4. Discussion

Based on the results from previous studies [18,19] and observed differences between large- and laboratory-scale fermentations, we expected to see increased pCO_2_ levels effect product and biomass yields at a lower level of enrichment than what was actually observed. The absence of any clear impact at 10% enrichment was surprising considering the measured pCO_2_ level in the large-scale fermentation was substantially lower than what this enrichment yielded throughout most of the fermentation (Figure 3). However, as the results reported by [18,19] were performed under very different physiological conditions with cultures grown in batch-mode and producing a protein instead of a metabolite, this could indicate that the impact of pCO_2_ is different depending on growth rate, and perhaps imposed production demand, medium composition, and nutrient availability. The observed differences in biomass and product yields between factory and laboratory even after CO_2_ enrichment thus provided a hint of other scale dependent factors being at work. It should be noted that we were not able to closely replicate the large-scale CO_2_ profile in the scale-down reactor as we could only enrich with a fixed percentage in the inlet gas stream. The large-scale vessel would therefore always have a more dynamic CO_2_ profile with larger differences between the peaks and troughs (Figure 3). Nonetheless, the pCO_2_ level in the large-scale fermentation was within the range encompassed by the 0% and 10% enrichments but for a few hours at the very peak in the early fermentation phase (Figure 3). While the 10% enrichment did not show a significant impact, this could be achieved by increasing the CO_2_ enrichment to 15% or 20%. While these enrichment levels resulted in a CO_2_ level that was higher than what was observed in our process, they were still within a range that is encountered in industrial operations [19].

A further increase of the CO_2_ enrichment up to 30% was also tested. This led to rapid acetic acid accumulation and a loss of culture viability shortly after the enrichment was initiated (Figure 6C,D). A likely explanation is that the high pCO_2_ level impacted the growth rate of the strain. The feeding profile used in this study was designed to avoid an accumulation of acetate from overflow metabolism [32,33]. However, if the µmax was significantly reduced by the elevated pCO_2_ levels, the threshold growth rate where acetate accumulation started was likely also reduced. Since the accumulation of acetate also reduces the µmax [32,34,35,36], this can quickly lead to a negative spiral with ever more acetate formation and growth rate reduction, eventually leading to a complete loss of the culture. This was precisely what was seen with very high base titration indicating that acid accumulation was already at the onset of the CO_2_ enrichment which continued to increase until the cultivation collapsed (Appendix C, Figure A1). The formation of high levels of acids was confirmed by an end point measurement of 32.5 g/L (542 mM) acetic acid, a level that is toxic to *E. coli* and highly inhibitory to growth [36,37]. This behavior also closely mimicked what we have observed when we increased feed rates in the past. These results together with reported results in the literature show that the precise onset of pCO_2_ growth inhibition is highly dependent on the organism and the growth conditions. Castan et al. reported a negative impact of 9.75% CO_2_ enrichment with *E. coli* K12, that was further reduced to 19.48%, whereas Baez et al. reported a positive impact at 20 mbar, which turned negative at 70 mbar also using a K12 strain [19]. The negative impact was then magnified when increasing the pCO_2_ level furter to 150 mbar and 300 mbar. In contrast, Knoll et al. surprisingly did not report any negative impacts on growth even when CO_2_ accumulated to a level of 800 mbar in an aerobic glycerol-limited fed-batch process under highly elevated pressure [21]. This pCO_2_ level was much higher than the maximum level of 260 mbar that was measured in the 30% enriched fermentations that led to a rapid culture loss with our process. The growth rate resulting from the feeding profile they used was also significantly higher following a feeding profile corresponding to a µ of 0.153 h^−1^, which should make the situation even worse. However, it should be noted that we have observed that the heavy burden of metabolic pathway overexpression and metabolite production can reduce the threshold growth rate where overflow metabolism starts quite substantially and increase the sensitivity towards runaway acetic acid caused culture failures (data not shown). This has necessitated the use of less aggressive feeding profiles in our process. A key difference was also that they used a stepwise increase in pressure and thereby pCO_2_ level and that their fermentation was much shorter. Indeed, they did observe a dramatic increase in osmotic pressure after 22 h together with an accumulation of mixed acids indicating that the growth would not be sustainable for long at this pressure and pCO_2_ level. 

Due to the particular traits of the process, this study also looked into the impact of sole carbon and C-N double limitation and how these limitations interacted with increased pCO_2_ levels. The potential for combining C-limitation with another nutrient limitation to redirect part of the carbon and energy consumption from the biomass into product formation is well known and can be an attractive choice for industrial production [38], [39]. Here, C/N-limited conditions were indeed shown to reduce biomass formation and increase product yield compared to C-limitation alone. In contrast to C/N-limitation, under C-limitation, no impact on biomass yield could be seen with the 15% pCO_2_ enrichment. This implied that the increased biomass in the 15% pCO_2_ enriched C/N-limited runs were indeed a result of increased nitrogen availability. The product yields for the C-, C/N- and, large-scale runs were also very close until approximately 30 h, which corresponded with the onset of N-limitation (Figure 6). After this point, the large-scale fermentation increased its carbon allocation to CO_2_ and decreased its allocation to the biomass and products. Thus, maintenance energy requirements were unanticipatedly increased. It is unknown whether this was caused by a change in physiology at the onset of C/N-limitation to one that was less well suited to the large-scale environment or if it coincided with a change in the mixing regime resulting in increased gradients in the large vessel as the volume increased and different impellers were engaged. In light of this result, it would be interesting to see if a relief of N-limitation could improve the yields post 30 h in large-scale fermentations.

The proteome analysis revealed that high pCO_2_ levels induced a greater number of differentially expressed proteins under C/N-limited conditions than C-limited. This was no surprise considering that the elevated CO_2_ level indirectly affected the degree of N-limitation by increasing the NH_4_OH titration, which was expected to have a major impact on the physiology. We did observe an increased GadBCE and GlsA glutaminase expression under both C-limited and C/N-limited conditions. Though the differential expression did not show up after filtering in the C-limited samples and only after 120 h in the C/N-limited fermentations. This response suggested that the intracellular pH was acidified by dissolved CO_2_. This has also been reported in other studies where CO_2_ triggered an acid response [40] or an increased GadABC expression level [18]. A general trend of higher expression values for TCA related proteins when exposed to CO_2_ enrichment was also observed for both C- and C/N-limited cultures. Since CO_2_ enrichment did not result in increased biomass formation under C-limitation, the higher TCA expression of especially GltA, Ppc, SdhAB, and SucABC was not likely a result of increased anaplerosis. However, it must be noted that reactions affected by high pCO_2_ levels would not necessarily lead to enzyme level changes.

A general trend of higher expression values for TCA related proteins when exposed to CO_2_ enrichment was also observed for both C- and C/N-limited cultures. This observation was the opposite of what Baez et al. found in their study, which showed lower carbon flux to TCA and reduced biomass yield under batch conditions. This was not unexpected as the growth rate in our fed-batch fermentations was much lower than the non-limited growth rate under batch conditions and was therefore not exhibiting overflow metabolism. Interestingly, the C-limited samples showed a higher expression of most TCA enzymes even in the control condition at all timepoints. A higher expression of SucABC in the CO_2_ enriched samples also suggested a higher flux in the TCA cycle and/or increased nitrogen assimilation. A higher TCA cycle flux could be related to increased Ppc (phosphoenolpyruvate carboxylase) activity, which has been observed to be upregulated in *Saccharomyces cerevisiae* under high CO_2_ concentrations [41]. Ppc fixates CO_2_ by carboxylation of the less reactive bicarbonate anion (HCO_3_-) in the cytoplasm to form oxaloacetate from phosphoenolpyruvate [42]. In addition to being upregulated in our dataset, its reaction would be favored by higher pCO_2_ levels and increase the supply of oxaloacetate to the TCA cycle.

Surprisingly, along with the upregulation of the L-malate dehydrogenase Mdh in the TCA cycle, a decarboxylating D-malate dehydrogenase DmlA was also upregulated, whereas MaeAB, a decarboxylating L-malate dehydrogenase, did not change its expression. The DmlA enzyme reduces D-malate to pyruvate and CO_2_ under anaerobic conditions and it is also involved in L-leucine biosynthesis [43]. Lukas et al. found that DmlA was essential for growth on D-malate under aerobic conditions and other C4-dicarboxylates were also seen to induce *dmlA* such as L- and meso-tartrate, while succinate did not trigger the expression [43]. From the data we have, we could not find an explanation for why it was induced under the tested conditions.

## 5. Conclusions

This study was designed to test how elevated pCO_2_ levels affect *E. coli* physiology and whether high pCO_2_ concentration observed in a very large industrial fed-batch process could account for the reduced product yield compared to the same process at the laboratory-scale. While it was not possible to obtain a close mimic of the pCO_2_ profile in the laboratory, it was observed that a process with 10% enrichment, which produced an equal or higher pCO_2_ level (of around 110 mbar) compared to the 450 m^3^ vessel throughout most of the process, did not affect the product formation. Therefore, other factors, alone or in combination with elevated pCO_2_, are required to account for the observed yield difference between the scales. The main candidate would be chemical gradients formed by the longer mixing times in the large vessel where particular variations in glucose concentration would be a prime suspect. However, increasing the pCO_2_ concentration beyond the level that was seen with the 10% enrichment by using 15% enrichment did impact product and biomass yields and increasing it further to 30% caused a full collapse of the culture. While both C and C/N limited cultures saw reductions in product yield, the CO_2_ enrichment only affected the biomass yield for the C/N-limited cultures indicating that this effect was due to a reduction of the degree of N-limitation. This was also reflected in the proteomics analysis which revealed surprisingly few changes for the C-limited condition. Here, the major differentially expressed proteins were in the TCA cycle, the two 2′-FL pathway related proteins Gmd and FutC, and the proteins involved in the acid stress response. Of these, changes to the TCA cycle and the 2′-FL pathway could potentially impact yields. However, the C-limited yield difference mainly manifested in the beginning of the fermentation when the proteomics differences in the 2′-FL pathway were very small. Thus, changes to the energetics, in which the TCA cycle plays a part, seems a more likely candidate.

## Figures and Tables

**Table 1:** Relative accumulated biomass and product yield on glucose.

CO_2_ Enrichment	Limitation	Number of Replicates	Relative Accumulated Biomass Yield (Yxs, %) *	Relative Accumulated Product Yield (Yps, %) *
0	C/N	3	100 ± 6.7	100 ± 8.3
10%	C/N	1	105	104
15%	C/N	3	106 ± 7	96 ± 2.0
20%	C/N	2	128–141	88–90
30%	C/N	2	n/a **	n/a **
0%	C	3	118 ± 5.1	85 ± 9.5
15%	C	2	115–116	89–92
**Large-scale**	**C/N**	3	**109 ± 4.7**	**77** **± 2.7**

* Summary of the results shown are either ranges from duplicate fermentations or average values calculated from triplicate fermentations with standard deviations. The values were relativized by normalizing to the average of the end-point value of the three fermentations without CO_2_ enrichment under C/N limitation. Yps includes 2′-FL and DFL. ** n/a: no stable run achieved under these conditions.

**Table 2:** Summary of differentially expressed proteins at various timepoints. Upregulation means higher expression in the CO_2_ enriched condition. Significant differential expression was defined as *p* < 0.05 and FC > 1.5.

C-Limited			C/N-Limited		
Comparison	Fermentation Phase	Number of DE Proteins	Comparison	Fermentation Phase	Number of DE Proteins
Control vs. 15% enriched	Early	22 (9↑,13↓)	Control vs. 15% enriched	Early	N/A
	Mid	17 (9↑,8↓)		Mid	139 (98↑,41↓)
	End	5 (3↑,2↓)		End	218 (163↑,55↓)

After the number of differentially expressed (DE) proteins, the number is specified into numbers of upregulated and downregulated (followed by ↑ and ↓, respectively)

## Data Availability

Restrictions apply to the availability of these data. The limited dataset that supports the findings of this study are available upon reasonable request from the authors and with the permission of Royal DSM.

## Appendix A

**Table A1:** Overview of the fermentations in the study. The number of replicate fermentations carried out for each condition is summarized as well as the number of fermentations used for the proteomics study. Sample timepoints for the proteomics study are listed.

CO_2_ Enrichment	Nutrient Limitation	Number of Replicate Fermentations	Number of Fermentations Sampled for Proteomics	Timepoints for Proteomics Sampling
Control (0%)	C/N	4	3	6 h
				30 h
				120 h
Control (0%)	C	3	2	6 h
				30 h
				80 h
				120 h
15% enriched	C	2	2	6 h
				30 h
				80 h
				120 h
10% enriched	C/N	1	1	30 h
				80 h
				120 h
15% enriched	C/N	3	2	6 h
				30 h
				80 h
				120 h
20% enriched	C/N	2	1	6 h
				30 h
				80 h
				100 h
30% enriched	C/N	2	n/a	n/a

## Appendix B

**Table A2:** The list of proteins included in the targeted search in the proteomics dataset. The list is divided into three functional groups: TCA cycle and pyruvate metabolism, 2FL production pathway, and Decarboxylation/carboxylation related proteins.

TCA Cycle/Pyruvate Metabolism			2′-FL Production	
Protein	Function		Protein	Function
AceB	malate synthase A		Gmd	GDP-mannose 4,6-dehydratase
AceK	isocitrate dehydrogenase kinase/isocitrate dehydrogenase phosphatase		LacY	lactose permease
AcnA	aconitate hydratase A		ManA	mannose-6-phosphate isomerase
AcnB	bifunctional aconitate hydratase B and 2-methylisocitrate dehydratase		ManB	phosphomannomutase
FumA	fumarase A		ManC	mannose-1-phosphate guanyltransferase
FumC	Fumarate hydratase		PtsG	glucose-specific PTS enzyme IIBC component
GltA	citrate synthase		FutC	fucosytransferase
Icd	Isocitrate dehydrogenase		WcaG/Fcl	GDP-L-fucose synthase
Lpd	lipoamide dehydrogenase		**Decarboxylation/Carboxylation**	
MaeA	NAD+-dependent malate dehydrogenase		Protein	Function
Mdh	malate dehydrogenase		Ppc	Phosphoenolpyruvate carboxylase
Ppc	Phosphoenolpyruvate carboxylase		AceF	Acetyltransferase, component of pyruvate dehydrogenase complex
SdhA	succinate:quinone oxidoreductase, FAD binding protein		PyrF	Orotidine 5′-phosphate decarboxylase
SdhB	succinate:quinone oxidoreductase, iron-sulfur cluster binding protein		NadC	Nicotinate-nucleotide pyrophosprolyase, decarboxylating
SdhC	succinate:quinone oxidoreductase, membrane protein		HemE	Uroporghyrinogen decarboxylase
SdhD	succinate:quinone oxidoreductase, membrane protein		Psd	Phosphatidylserine decarboxylase proenzyme
SucA	2-oxoglutarate decarboxylase, thiamine-requiring		SucB	succinyltransferase component of 2-oxoglutarate dehydrogenase
SucB	dihydrolipoyltranssuccinylase		SucA	2-oxoglutarate decarboxylase
SucD	succinyl-CoA synthetase subunit α		LpdA	lipoamide dehydrogenase
			Icd	isocitrate dehydrogenase
			PoxB	Pyruvate oxidase
			MaeA	malate dehydrogenase, oxaloacetate-decarboxylating and NAD+ dependent
			MaeB	malate dehydrogenase, oxaloacetate-decarboxylating and NADP+ dependent
